# *In-silico* identification of the vaccine candidate epitopes against the Lassa virus hemorrhagic fever

**DOI:** 10.1038/s41598-020-63640-1

**Published:** 2020-05-06

**Authors:** Prabin Baral, Elumalai Pavadai, Bernard S. Gerstman, Prem P. Chapagain

**Affiliations:** 1 Department of Physics, Florida International University, Miami, Florida 33199 USA; 2 Biomolecular Science Institute, Florida International University, Miami, Florida 33199 USA; 3 Present Address: Department of Physiology and Biophysics, Boston University School of Medicine, Boston, MA 02118 USA

**Keywords:** Viral infection, Biophysics, Computational biology and bioinformatics, Drug discovery, Immunology, Molecular biology

## Abstract

Lassa virus (LASV), a member of the *Arenaviridae*, is an ambisense RNA virus that causes severe hemorrhagic fever with a high fatality rate in humans in West and Central Africa. Currently, no FDA approved drugs or vaccines are available for the treatment of LASV fever. The LASV glycoprotein complex (GP) is a promising target for vaccine or drug development. It is situated on the virion envelope and plays key roles in LASV growth, cell tropism, host range, and pathogenicity. In an effort to discover new LASV vaccines, we employ several sequence-based computational prediction tools to identify LASV GP major histocompatibility complex (MHC) class I and II T-cell epitopes. In addition, many sequence- and structure-based computational prediction tools were used to identify LASV GP B-cell epitopes. The predicted T- and B-cell epitopes were further filtered based on the consensus approach that resulted in the identification of thirty new epitopes that have not been previously tested experimentally. Epitope-allele complexes were obtained for selected strongly binding alleles to the MHC-I T-cell epitopes using molecular docking and the complexes were relaxed with molecular dynamics simulations to investigate the interaction and dynamics of the epitope-allele complexes. These predictions provide guidance to the experimental investigations and validation of the epitopes with the potential for stimulating T-cell responses and B-cell antibodies against LASV and allow the design and development of LASV vaccines.

## Introduction

Lassa virus (LASV), a member of the *Arenaviridae*^1^, is an ambisense RNA virus that causes a severe hemorrhagic Lassa fever in humans. LASV is endemic, particularly in the West African countries of Sierra Leone, The Republic of Guinea, Nigeria, and Liberia^2,3^. The transmission of LASV to humans occurs through the urine or feces of infected Mastomys rats and the virus spreads human-to-human through direct contact with the blood, urine, feces, or other bodily secretions of an infected person. LASV can be fatal and no approved effective therapeutics are currently available. The development of therapeutics such as antibodies and vaccines for the treatment of LASV is therefore of significant urgency^4–6^.

**Figure 1:**
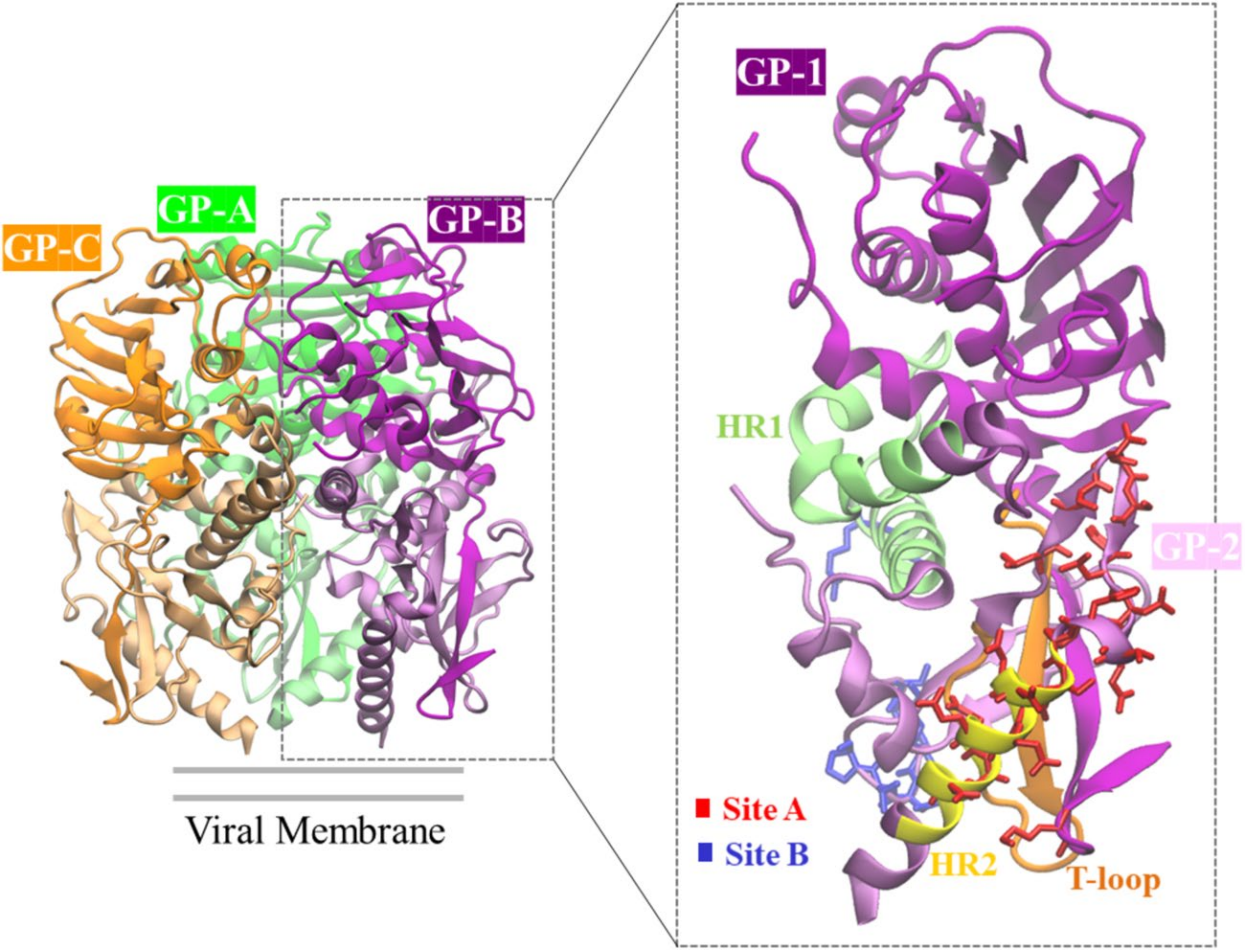
3D structure of the LASV GP trimer consisting of the three GPs (GP-A, GP-B, GP-C). Each GP has a GP1 subunit and a GP2 subunit (zoomed view). Each monomer is colored differently in the GP trimer. In the zoomed view, the GP2 subunit is lightly shaded to differentiate from the GP1 subunit, and some of the antibody binding sites (Site A, Site B) are highlighted (figure generated from the crystal structure of the LASV GP in the Protein Data Bank^21^, PDB ID: 5VK2^4^).

Of the four proteins that are encoded by the two RNA segments of the LASV genome, the glycoprotein (GP) is the only protein on the viral surface. GP results from the cleavage of a 75 kDa precursor polypeptide, GPC by signal peptidase and then further glycosylated and processed into GP1 and GP2^7^. GP1 is the receptor-binding subunit, and GP2 is the membrane-spanning fusion subunit^8–10^. The virion envelope protein spikes are composed of three heterotrimers, with each heterotrimer containing signal peptide, GP1, and GP2^11,12^, shown in Fig. 1. A chalice-like GP trimer interacts with receptors on the cell surface, for example matriglycan, which mediates the entry of the virus into the host cell. In addition, the GP also interact with ERGIC-53 in the exocytic pathway, which helps to form infectious virions^13^. GP is considered to be a key factor for LASV growth, cell tropism, host range and pathogenicity, and as it is the only protein situated on the LASV virion surface, GP becomes a primary target for vaccine design^4^.

The crystal structure of the trimeric LASV GP in complex with the 37.7 H neutralizing antibody from a human survivor (PDB ID: 5VK2, Fig. 1) has been determined, thereby providing insight into the structural basis for antibody design. Analysis of the GP-37.7 H antibody complex shows that the antibody simultaneously binds to two GP monomers at the base of the GP trimer. The binding involves four discontinuous regions of LASV GP: two in site A and two in site B. Site A contains residues 62 and 63 of the N-terminal loop of GP1 and residues 387 to 408 in the T-loop (residues 365–384) and HR2 (residues 400–412) regions of GP2. Site B contains residues 269 to 275 of the fusion peptide and residues 324 to 325 of HR1 (residues 311–355) of GP2^4,14^. Although the antibody predominantly binds to GP2, GP1 is required to maintain the proper prefusion conformation of GP2 for antibody binding^4^.

Identification of epitopes is an essential step for understanding disease etiology, immunotherapy, immunodiagnostics, and the discovery and development of epitope based-vaccines. An epitope-based vaccine has fewer side effects compared to conventional vaccines. Experimental identification of a promiscuous epitope involves many expensive and time-consuming steps, including the production of antibodies to map antigenic regions on a target protein, animal models, and determination of the crystal structure of antigen-antibody complexes using X-Ray crystallography. Computational identification of epitopes is often employed as a powerful and fast approach to facilitate the identification of potential epitope candidates that can decrease the number of validation experiments and time^15,16^. Multi-epitope based vaccine development has already proven effective against several viral infections and cancer^17,18^. In this study, we have identified and characterized T and B-cell epitopes for the LASV GP using different sequence and structure-based computational epitope prediction methods. We then selected potential B and T-cell epitopes for the LASV GP based on a consensus approach, and the novelty of the epitopes was examined with the Immune Epitope Database (IEDB) tools. Subsequently, we identified strongly binding alleles to the MHC-I T-cell epitopes and modeled the allele structures and performed docking to understand the interaction between alleles and epitopes. We further investigated the stability and dynamics of the epitope-allele complexes using molecular dynamics simulations. Analyses of root-mean square deviations, hydrogen bond, interaction energy, and solvent accessibility showed that epitope-allele complexes are stable, indicating that the epitopes strongly bind to the alleles. The identified B and T-cell epitopes of LASV GP in the study can be useful for the development of effective vaccines against Lassa hemorrhagic fever.

## Materials and methods

### Selection of LASV GP sequence and 3D structure

The sequence of GP for different LASV strains was obtained from the NIAID Virus Pathogen Database and Analysis Resource (ViPR)^19^. Subsequently, multiple sequence alignments were performed between the sequences using Clustal Omega^20^ to select a conserved LASV GP for sequence-based epitope prediction. The corresponding X-Ray crystal structure of the Mouse/Sierra Leone/Josiah/1976 LASV GP was obtained from the Protein Data Bank (PDB ID: 5VK2)^4,21^ for structure-based B-cell epitope prediction. The missing residues were modeled using the Charmm-Gui^22–24^.

### Prediction of B-cell epitopes

Sequence-based B-cell epitope prediction was performed with the use of BepiPred2.0^25^, BCPREDS^26^ and BcePred^27^ servers separately. These servers predict epitopes based on physico-chemical properties of amino acids, and these servers accept the primary sequence of LASV GP as an input.

Structure-based B-cell epitope prediction for the LASV GP (PDB ID: 5VK2) was carried out using three different programs separately: ElliPro^28^, Epitopia^29^ and DiscoTope^30^. These servers predict epitopes regions based on the geometrical and solvent surface-accessibility of a protein structure, and these servers accept the 3D structure of a protein as input. The consensus epitopes from both sequence and structure-based predictions were selected as potential epitopes for further analysis.

### Prediction of T-cell epitopes

Sequence-based MHC-I T-cell epitope predictions for LASV GP were carried out by using three different servers, ProPred-I^31^, CTLPred^32^ and NetCTL1.2^33^. To predict their alleles, the consensus epitopes among these three prediction methods were analyzed using IEDB^34^. The epitopes that strongly bind to the alleles (lowest IC_50_) were selected for further analysis.

Sequence-based MHC-II T-cell epitope predictions for LASV GP were performed with the use of three different servers: ProPred^35^, NetMHCII2.3^36^ and EpiTOP3.0^37^. The antigenicity score of the selected epitopes was predicted by VaxiJen 2.0^38^.

### Homology modeling and epitope-allele docking

The structure of HLA-A*02:06 (A1) [PDB ID 3OXR^39^], HLA-A*02:03 (A2) [PDB ID: 3OX8^39^], and HLA-B*35:01 (A3) [PDB ID: 2CIK^40^] were obtained from the PDB. The experimental structure for the HLA-A*32:01 (A4) allele is not available, and thus, the sequence of this allele was obtained from the UniProt database (UniProtKB ID: P01892), and subsequently its structure was modeled using Swiss-Model^41–43^. The selected consensus MHC-1 epitopes were extracted from the crystal structure of LASV GP (PDB: 5VK2). The epitopes and the alleles were prepared for docking using Autodock Tool version 1.5.6^44^. Autodock Vina 1.1.2^45^ was used for peptide docking with a grid space that covered the entire allele. The best peptide-allele complexes were selected for further investigation based upon visual inspection of peptide-allele interactions and the Autodock Vina criteria. The stability and dynamics of the selected peptide-allele complexes were further studied using molecular dynamics simulations.

### Molecular dynamics simulations

All-atom, explicit-solvent molecular dynamics (MD) simulations were performed to investigate the stability and dynamics of the MHC-1 T-cell epitope-allele complexes using the CHARMM36m force field^46^ with the NAMD 2.12 software package^47^. The systems were minimized for 10,000 steps followed by 200 ps of equilibration. This was followed by MD production runs for 200 ns at a temperature of 300 K using a 2 fs time-step. The long-range ionic interactions were calculated using the particle mesh Ewald (PME) method^48^ while the covalent hydrogen atoms were constrained by using a SHAKE algorithm^49^. The temperature was controlled by using the Langevin temperature coupling with a friction coefficient of 1 ps^−1^ and pressure was controlled using the Nose-Hoover Langevin-Piston method^50^. Visualization, and rendering of trajectories and pictures were performed using VMD^51^.

## Results and Discussion

**Figure 2:**
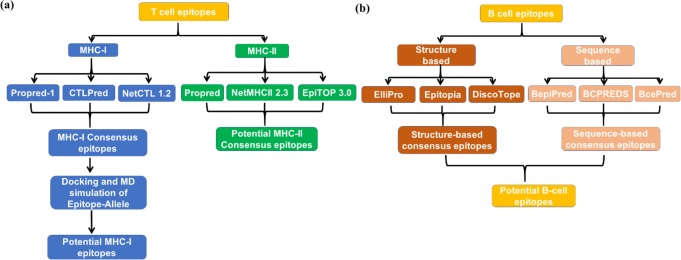
The workflow cascade for epitope identification of (**a**) T-cell and (**b**) B-cell.

The multiple sequence alignment of the 84 LASV GP sequences resulted in the LASV GP Mouse/Sierra Leone/Josiah/1976) [UniprotKB ID: P08669] as a highly conserved strain, and we thus selected this strain for the sequence-based MHC-I and MHC-II T-cell epitope predictions and for both structure and sequence-based B-cell epitope predictions. In addition, a search of this strain with the experimentally determined structure available in the PDB displayed the 3.2 Å resolution crystal structure of the prefusion GP trimer of LASV in complex with the human neutralizing antibody 37.7 H. [PDB ID: 5VK2] as shown in Fig. 1. This structure was used for the structure-based B-cell epitope prediction. A schematic representation of the epitope prediction cascade is shown in Fig. 2. We have adopted multiple methods to predict and rank the epitopes as they use different criteria for their predictions. Some approaches may incorporate some properties that are similar such as solvent accessible surface area, but the predicted epitopes are different. Previous studies^52,53^ have suggested that the consensus approach would improve the specificity and accuracy of the epitope prediction as it can reduce the false positives. Therefore, we employ a consensus approach; for example, an epitope can be considered if it overlaps with even a single residue by at least two prediction methods. Our consensus approach selected several nanomer epitopes for MHC-I (Table S1). Although the predicted epitopes for MHC-II T-cell vary in length, the consensus core region between predicted MHC-II epitopes is a nanomer (Table S2) which is considered^54^ an optimal length for the HLA.

### Prediction of T-cell Epitopes

MHC-I T-cell epitope prediction with the LASV GP sequence was performed using three different methods separately: ProPred-1, CTLPred, and NetCTL1.2, and the results are shown in Supplementary Table S1. The epitopes listed by at least two of the methods are listed in Table 1 along with their binding affinity (IC_50_), antigenicity, and allele. Among these four consensus epitopes, the nanomer E1 epitope FATCGLVGL shows the lowest average IC_50_ value of 34 nM against the A1 allele as predicted by the IEDB, and it has also a reasonable antigenicity score of 1.65. This was followed by the E3 epitope FSRPSPIGY, which has an average IC_50_ value of 88 nM against the A3 allele, and also has a better antigenicity score of 2.50 compared to the FATCGLVGL epitope. Interestingly, the E4 epitope RRGTFTWTL is predicted by all three methods though its IC_50_ and antigenicity scores are not as good as the other epitopes (Table 1). All four of these consensus epitopes were docked to the alleles and we performed the MD simulations to investigate the stability and dynamics of the allele-epitope complex as discussed later.

**Table 1:** Consensus prediction of the MHC-I T-cell epitopes.

Epitope	Sequence	Interval	Prediction method			Antigenicity	IC_50_ (nM)		Allele
			ProPred-1	CTLPred	NetCTL 1.2		ANN	SMM	
E1	FATCGLVGL	38–46	✓	✓	×	1.65	11.91	55.79	HLA-A*02:06 (A1)
E2	IINHKFCNL	112–120	✓	✓	×	1.23	101.6	214.8	HLA-A*02:03 (A2)
**E3**	FSRPSPIGY	233–241	×	✓	✓	2.50	81.63	94.1	HLA-B*35:01 (A3)
E4	**RRGTFTWTL**	**258–266**	✓	✓	✓	**1.04**	**109.6**	**727.7**	HLA-A*32:01 (A4)

The epitope predicted by all three methods is highlighted in boldface.

MHC-II T-cell epitope prediction with the LASV GP sequence was performed using three different methods separately: ProPred, NetMHCII 2.3, and EpiTOP 3.0, and the results are shown in Supplementary Table S2. ProPred uses a quantitative matrix^35^ approach and NetMHCII2.3 uses ANN^36^, while EpiTOP 3.0 uses Quantitative Structure–Activity Relationship models (QSAR)^37^ to predict the MHC-II T-cell epitopes. The epitopes that were predicted by at least two methods are listed in Table 2. Among these consensus MHC-II T-cell epitope predictions, the E9 and E13 epitopes were predicted by all three methods and have a reasonable antigenicity score of 0.7, indicating that these two epitopes can be potential candidates for the design of MHC-II T-cell based vaccines. ProPred and EpiTOP 3.0 predict most epitopes as nanomers whereas NetMHCII 2.3 predicts varying lengths of epitopes (Table 2). Interestingly, the 15-mer epitopes predicted by NetMHCII have the consensus core nanomer epitopes, suggesting that the core region is responsible for strong binding of the epitope into the MHC-II binding site^55–57^.

**Table 2:** Prediction of the MHC-II T-cell epitopes.

Epitope	Sequence	Interval	Prediction Method			Antigenicity
			ProPred	NetMHCII 2.3	EpiTOP 3.0	
E5	MGQIVTFFQ	1–9	✓	×	✓	−0.1820
E6	VYELQTLEL	65–73	✓	×	✓	0.8600
E7	LNMTMPLSC	78–86	✓	×	✓	0.9390
E8	INHKFCNLS	113–121	✓	×	✓	1.5060
**E9**	***MSIISTFHL***	**134–142**	✓	✓	✓	**0.7080**
	LYDHAL**MSIISTFHL**	128–142	×	✓	×	0.2896
	YDHAL**MSIISTFHLS**	129–143	×	✓	×	0.4907
	DHAL**MSIISTFHL**SI	130–144	×	✓	×	0.4809
	HAL**MSIISTFHL**SIP	131–145	×	✓	×	0.1949
	AL**MSIISTFHL**SIPN	132–146	×	✓	×	0.2066
	L**MSIISTFHL**SIPNF	133–147	×	✓	×	0.2428
E10	FNQYEAMSC	147–155	✓	×	✓	0.5520
E11	ISVQYNLSH	162–170	✓	×	✓	1.1310
E12	LQTFMRMAW	188–196	✓	✓	×	0.2620
	VANGV**LQTFMRMAW**G	183–197	×	✓	×	0.1328
	ANGV**LQTFMRMAW**GG	184–198	×	✓	×	0.1683
	NGV**LQTFMRMAW**GGS	185–199	×	✓	×	0.0579
	GV**LQTFMRMAW**GGSY	186–200	×	✓	×	0.1572
	V**LQTFMRMAW**GGSYI	187–201	×	✓	×	0.1895
**E13**	***MRMAWGGSY***	**192–200**	✓	✓	✓	**0.7630**
	GVLQTF**MRMAWGGSY**	186–200	×	✓	×	0.1572
	VLQTF**MRMAWGGSY**I	187–201	×	✓	×	0.1895
	LQTF**MRMAWGGSY**IA	188–202	×	✓	×	0.1902
	QTF**MRMAWGGSY**IAL	189–203	×	✓	×	0.3470
	TF**MRMAWGGSY**IALD	190–204	×	✓	×	0.4434
	F**MRMAWGGSY**IALDS	191–205	×	✓	×	0.3543
E14	YQYLIIQNT	217–225	✓	✓	×	0.4720
	DCIMTS**YQYLIIQNT**	211–225	×	✓	×	0.6600
	CIMTS**YQYLIIQNT**T	212–226	×	✓	×	0.7075
	IMTS**YQYLIIQNT**TW	213–227	×	✓	×	0.6029
	TS**YQYLIIQNT**TWED	215–229	×	✓	×	0.6874
E15	LIIQNTTWE	220–228	✓	×	✓	0.9100
E16	IGYLGLLSQ	239–247	✓	×	✓	1.5300
E17	LLSQRTRDI	244–252	✓	×	✓	1.7310
E18	IYISRRRRG	252–260	✓	✓	×	1.5560
	SQRTRD**IYISRRRRG**	246–260	×	✓	×	1.6434
	QRTRD**IYISRRRRG**T	247–261	×	✓	×	1.5276
	RTRD**IYISRRRRG**TF	248–262	×	✓	×	1.7213
	TRD**IYISRRRRG**TFT	249–263	×	✓	×	1.4112
	RD**IYISRRRRG**TFTW	250–264	×	✓	×	1.5207
	D**IYISRRRRG**TFTWT	251–265	×	✓	×	1.4261
	**IYISRRRRG**TFTWTL	252–266	×	✓	×	1.2680
E19	WMLIEAELK	283–291	✓	×	✓	1.3250
E20	IQLINKAVN	334–342	✓	×	✓	0.7710
E21	LINDQLIMK	344–352	✓	×	✓	−0.0481
E22	LRDIMCIPY	355–363	✓	×	✓	1.0590
E23	LVSNGSYLN	387–395	✓	×	✓	0.3450

The epitopes predicted by all three methods are highlighted in boldface with Italic font. The consensus core regions highlighted in boldface are in the epitopes predited by NetMHCII 2.3.

### Prediction of B-cell epitopes

In addition to the T-cell epitope predictions, we also predicted the linear B-cell epitopes for the LASV GP using sequence-based methods BepiPred 2.0^25^, BCPREDS^26^, and BcePred^27^. The BepiPred predicts the epitopes based on a random forest algorithm trained on epitopes annotated from antibody-antigen structures. BCPREDS predicts epitopes by using SVM combined with a different kernel method, including string kernels, radial basis kernels, and subsequence kernels. The BcePred locates B-cell epitopes using four physicochemical properties like hydrophilicity, polarity, exposed surface and beta-turns^27^. The epitope E30 containing 10 residues was predicted by all three of these sequence methods (Table 3) but with a negative antigenicity score.

**Table 3:** Prediction of the B-cell epitopes.

Epitope	Sequence	Interval	Sequence based			Structure based			Rank	Antigenicity
			BepiPred	BCPREDS	BcePred	ElliPro	Epitopia	DiscoTope		
**E24**	***LSDAHKKNLYD***	**120–130**	✓	×	✓	✓	✓	✓	**5/6**	**0.74**
E25	PNFNQYEA	145–152	✓	×	✓	✓	✓	×	4/6	0.4565
E26	DFNGGKI	156–162	×	✓	×	✓	✓	×	3/6	0.7315
E27	LSHSYAGDAANHCGT	168–182	✓	×	×	✓	✓	×	3/6	0.0814
E28	LDSGCGNWDCIMTSYQY	203–219	×	✓	×	✓	✓	×	3/6	1.0802
**E29**	***ISRRRRGT***	**254–261**	×	×	✓	✓	✓	✓	**4/6**	**1.2517**
**E30**	**SDSEGKDTPG**	**267–276**	✓	✓	✓	✓	✓	×	**5/6**	**−0.0739**
E31	NHTTTGRT	373–380	×	✓	✓	✓	✓	×	4/6	0.9941
**E32**	***ETHFSDDIE***	**396–404**	✓	×	✓	✓	✓	✓	**5/6**	**0.4989**
**E33**	***MLQKEYMERQ***	**414–423**	×	✓	✓	✓	✓	✓	**5/6**	**−0.14**

The epitopes predicted by either all three sequence- or structure-based methods are highlighted by boldface. Conformational epitopes chosen by all three structure-based methods are indicated in italics.

We also performed structure-based B-cell epitope prediction using three representative structural and geometrical properties-based methods: ElliPro, Epitopia and DiscoTope. For this, the experimental 3D structure LASV GP (PDB ID: 5VK2) with the modeled missing residues was used. ElliPro predicts linear and conformational epitopes by incorporating the antigenicity, solvent accessibility, and flexibility of protein structures^28^. Epitopia uses a machine learning algorithm to analyze the antigenic features on protein structure and predicts the probable conformational epitope regions^29^. DiscoTope uses amino acid statistics, spatial information, and surface accessibility on the protein 3D structure to predict residue-by- residue conformational epitopes^30^. The E24, E29, E32 and E33 structure-based epitopes in Table 3 are especially interesting as potential candidates as they were predicted by all three methods. In Table 3, we also ranked each epitope based upon how many of the sequence and structure-based methods predicted each epitope, which do not always correlate with the highest antigenicity scores of E24, E26, E28, E29 and E31.

Robinson *et al*.^14^ have recently reported the cloning of many human monoclonal antibodies derived from memory B cells of Lassa fever survivors in West Africa. These antibodies specifically bind to both GP1 and GP2 epitopes of LASV. The comparison of our predicted B-cell epitopes with those epitopes shows that there are five consensus epitopes (Table 3) that share similarity with Robinson *et al*. (Table S3), and another five epitopes that do not share similarity, indicating that our consensus epitope prediction strategy has identified new epitopes.

### Epitope surface mapping

**Figure 3:**
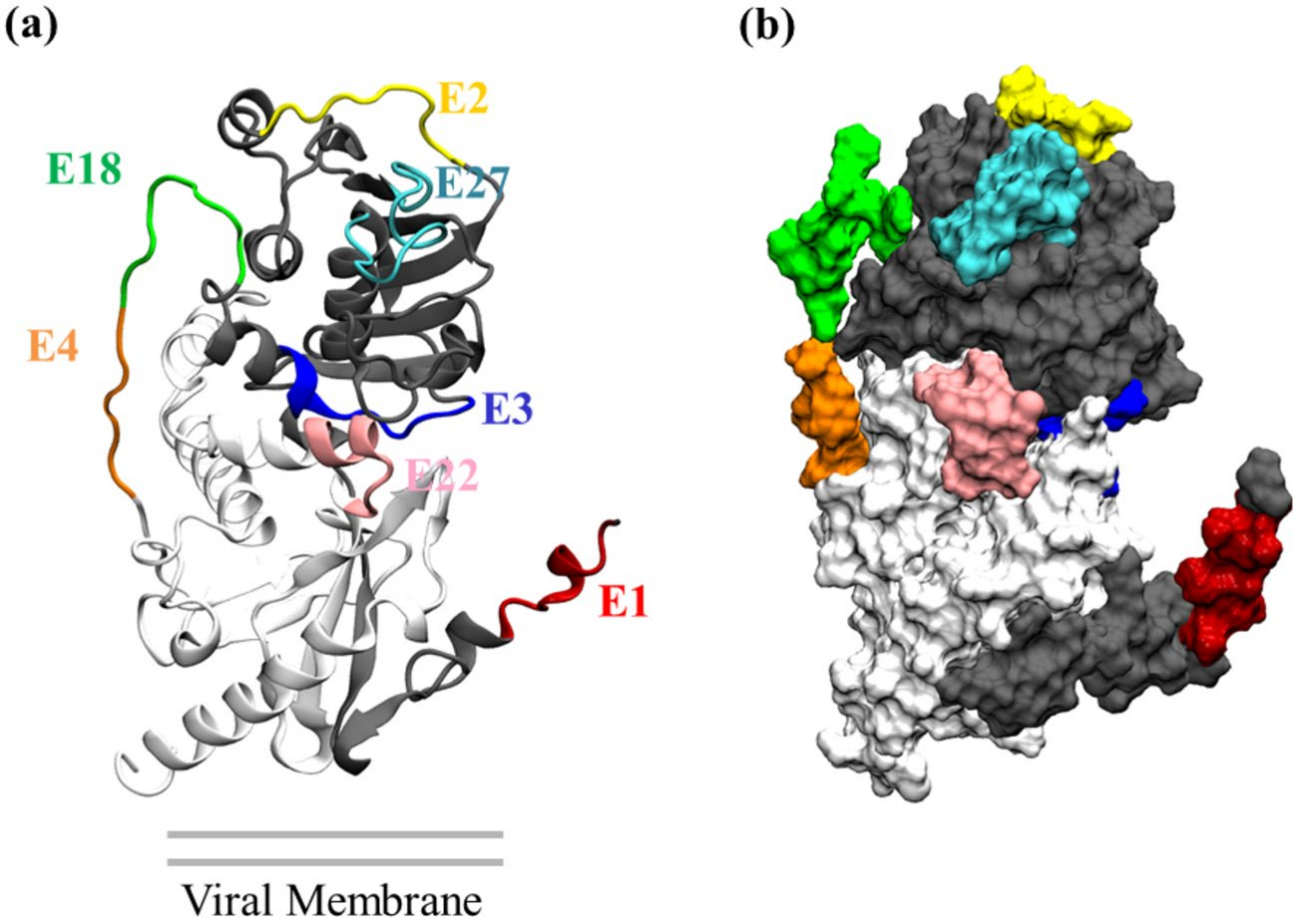
Mapping of some representative epitopes are highlighted on the LASV GP. Mapping of: (**a**) secondary structural elements, (**b**) surface accessibility. The location of the epitopes on the GP suggests that they are on the solvent exposed region, indicating promiscuity as they have easy access to alleles.

For efficacy of vaccines, the epitopes should be located on an accessible region of the protein so that the epitope will be able to bind with antibodies^53^. This is especially important for the six epitopes that we list in the Tables above that do not share any part of their sequence with known epitopes: E1, E4, E18, E22, E27, E29. In Fig. 3, we highlight the positions of these epitopes on LASV GP. We also highlight the positions of E2 and E3 because the four MHC-I T-cell epitopes have IC_50_ information readily available. Figure 3 shows that the E1, E2, E3, E4, E18, E22 and E27 epitopes are well located on the exposed regions and thus can interact well with the alleles.

### MHC-I T-cell Allele and epitope modeling and docking

Swiss-Model identified the 1.61 Å resolution crystal structure of the HLA class I antigen (PDB ID: 6EI2) as the best template for constructing models. The sequence identity between A4 and the template was 92%. The best model was then selected based on multiple validation methods, including GMQE (Global Model Quality Estimation) and QMEAN. The GMQE and QMEAN values^41,58^ of the model are 0.75, and 0.6, respectively. In addition to these analyses, Ramachandran plots and ERRAT were also used for the model validation. Analysis of Ramachandran plot^59^ of the model shows 99.6% of residues are either in favored or in allowed regions (Supplementary Fig. S1), indicating that backbone torsion angles of these models are acceptable. The ERRAT overall quality factor^60^ score was computed as 99, which is greater than the normally accepted score range for a high quality model of 50. These analyses show that the model is within a high quality range and can be used for further analysis.

**Figure 4:**
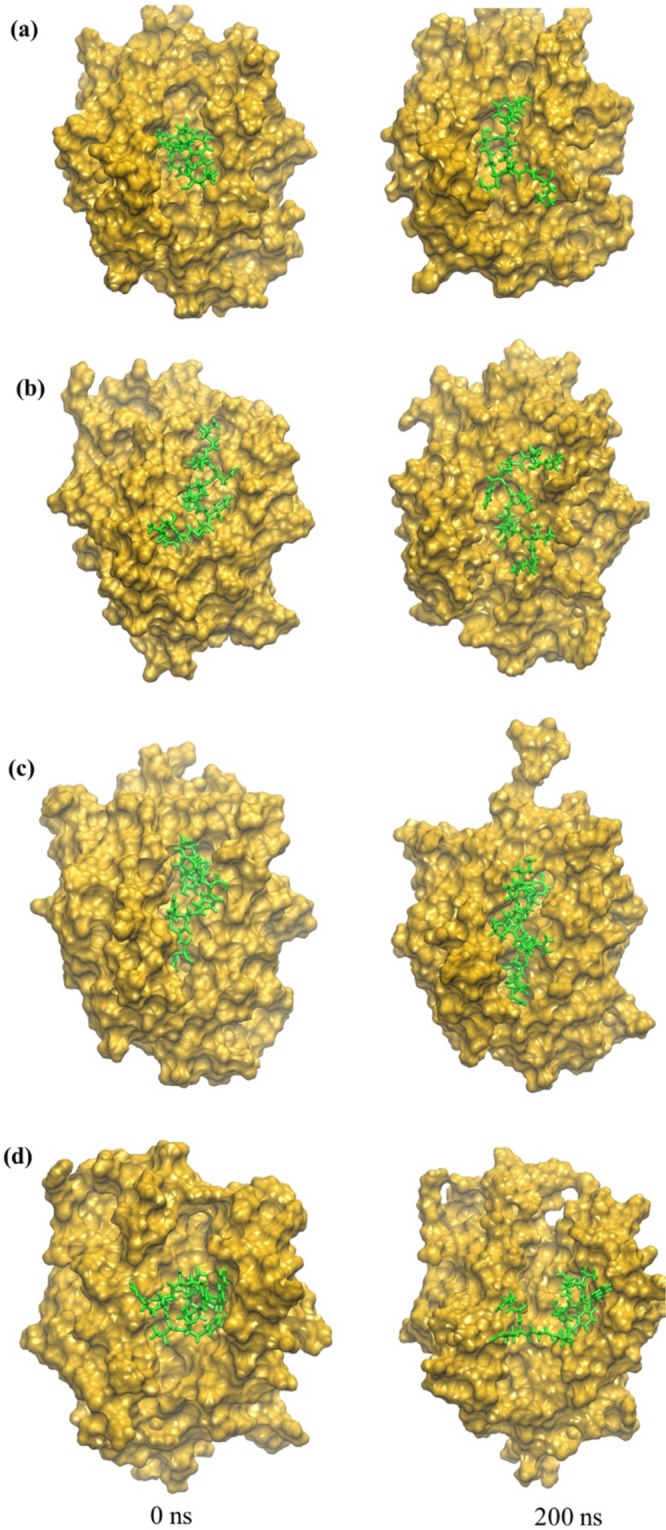
Snapshots of allele-epitope complexes. (**a**) A1::E1, (**b**) A2::E2, (**c**) A3::E3, and (**d**) A4::E4 at the beginning and end of the MD simulations: t = 0 (minimized structure), t = 200 ns. Allele is gold and epitope is green.

Docking of the four consensus MHC-I epitopes (Table 1) was performed using Autodock Vina, which enabled the docking of epitopes obtained from the sequence-based MHC-1 T-cell prediction into the promising allele structures. The Autodock Vina docking protocol has been previously demonstrated to successfully dock epitopes into allele structures^45^. However, we validated the capability of the docking protocol before docking the epitopes by redocking the epitopes into the allele crystal structure (PDB ID:3OX8) to see whether the crystal bound conformation of the peptide could be reproduced or not. The docked allele-epitope complex showed the same residue-epitope interactions observed in the epitope bound crystal structure, indicating that the Autodock Vina docking protocol was capable of reproducing the experimentally observed binding mode of the epitope. We applied Autodock Vina to each of the four MHC-I allele-epitope complexes. Autodock Vina found that the highest ranked docking structure had the following binding affinities: −5.5 kcal/mol for A1::E1 −5.0 kcal/mol for A2::E2, −6.8 kcal/mol for A3::E3, and −6.0 for A4::E4. These epitopes-alleles docking complexes are shown in Fig. 4.

### Dynamics of the allele-epitope complex

**Figure 5:**
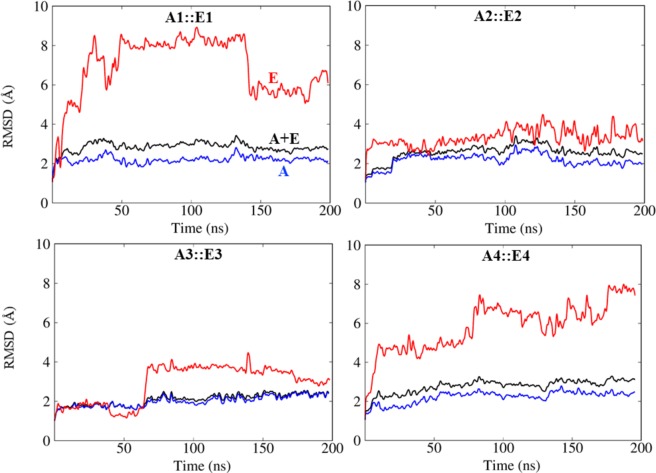
Root-mean-squared deviations (RMSD) calculated for the backbone atoms of allele (A), epitope (E) and complex (A + E) from MD simulations of MHC-I allele-epitope complexes.

In order to investigate the dynamics and stability of the four MHC-I allele-epitope complexes, we performed 200 ns all-atom, explicit solvent MD simulations. To quantitatively understand the stability of the allele-epitope complex, we calculated the root mean square deviations (RMSD) of the backbone atoms of the allele-epitope complexes as a function of simulation time as shown in Fig. 5. Figure 5 also includes curves of the RMSD of the backbone atoms of just the allele, and separately, just the backbone atoms of the epitope. All alleles have an RMSD compared to their initial structures of approximately 2 Å, whereas the allele-epitope complexes have a bit higher RMSD of approximately 2.5 Å, indicating that the epitopes make the complexes more flexible. Interestingly, in the case of A3::E3, the allele and the complex show almost the same RMSD, suggesting that the complex is especially stable. To pinpoint why the complexes show a higher RMSD, we further computed the RMSD of only the backbone atoms of the epitope in each the complex. Figure 4 shows that the initial configuration of epitopes E1 and E4 is compact, and that both of these epitopes rearrange their configuration in the binding site and elongate during the 200 ns MD simulation. This elongated configuration is consistent with the investigations of Antunes *et al*.^61^ on MHC-I epitopes.

**Figure 6:**
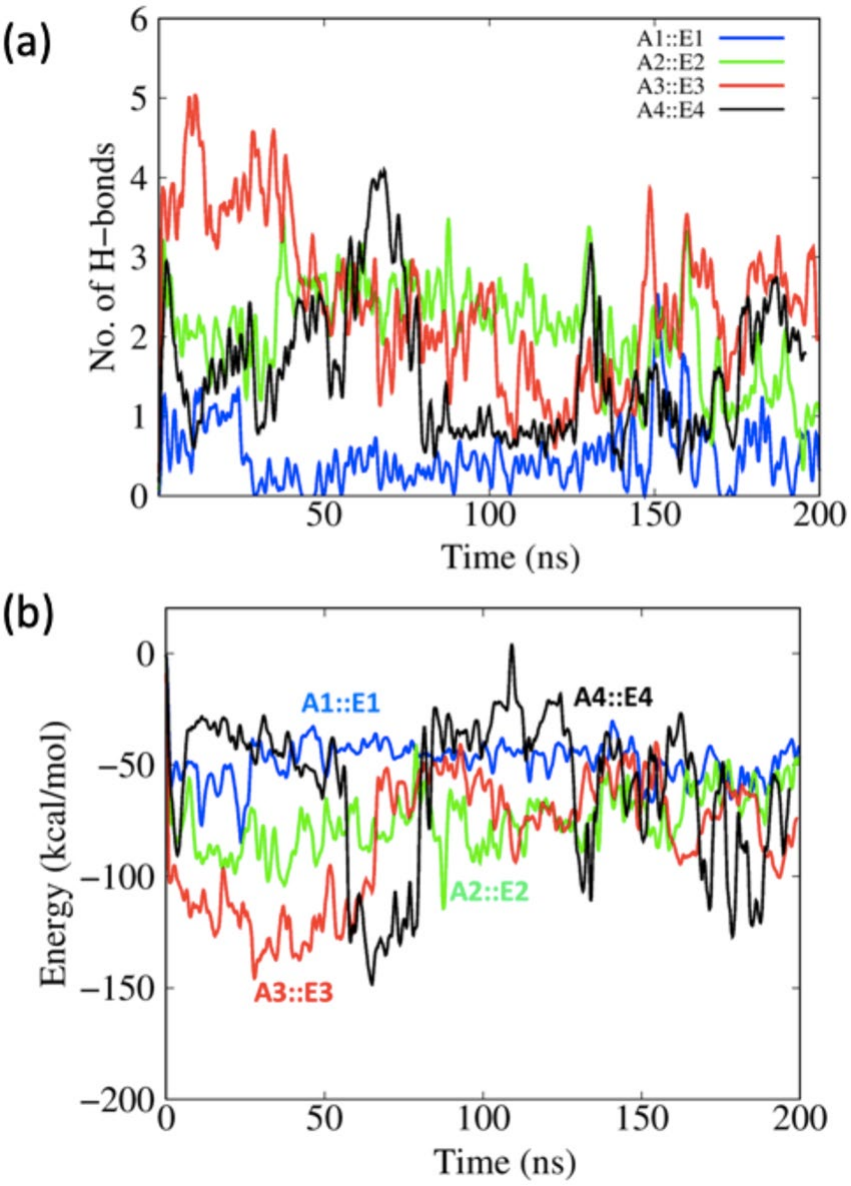
(**a**) The number of allele-epitope intermolecular hydrogen bonds as a function of MD simulation time. (**b**) Interaction energy calculated between allele and epitopes as a function of simulation time.

Since the interactions between protein and epitope peptide are mostly influenced by non-covalent interactions, we computed the number of hydrogen bonds and the interaction energy between the allele and epitope as a function of the MD simulation time. The hydrogen bond was calculated between the protein interface atoms with a distance cut-off of 3.5 Å and angle cut-off of 30o between the donor and acceptor heavy atoms. As shown in Fig. 6, the number of H-bonds fluctuates during the MD simulations for all the complexes. The A3 complex has the largest number of H-bonds. Table 4 shows that during the last 50 ns of the MD simulation trajectory, the A3 complex averages 2.5 H-bonds. Additional analysis of the hydrogen bonding between allele and epitope are listed in Supplementary Table S4.

**Table 4:** Allele–epitope interaction parameters calculated by averaging over the last 50 ns of the MD simulation trajectory.

Complex	Interaction Energy (kcal/mol)	No. of H-bonds
A1::E1	−53.53 ± 7.40	0.64 ± 0.54
A2::E2	−64.54 ± 10.88	1.49 ± 0.63
**A3::E3**	**−74.85 ± 14.94**	**2.48 ± 0.50**
A4::E4	−73.23 ± 27.07	1.51 ± 0.67

Figure 6b shows the interaction energy (electrostatic interaction + van der Waals contacts) throughout the entire MD simulation and Table 4 lists the average over the last 50 ns. The A3::E3 and A4::E4 display relatively stronger interaction energies than the A1:E1 and A2::E2 complexes. The comparison of RMSD, hydrogen bond, and interaction energy information indicates that the E3 epitope is an especially promising epitope candidate.

### Novelty analysis

The novelty of the four MHC I T-cell epitopes in Table 1, the nineteen MHC II T-cell epitopes in Table 2, and the ten B-cell epitopes in Table 3 identified in this study were analyzed using IEDB^34^. The IEDB database contains the epitopes that are annotated based on scientific literature. The IEDB showed that the E1, E4, E18, E22, E27, E29 epitopes, which bind to solvent exposed regions on the protein (Fig. 3), have not been previously reported as LASV epitopes or vaccine candidates. In addition, this analysis further indicates that 24 other epitopes (E2, E3, E5, E6, E7, E8, E10, E11, E12, E14, E15, E16, E17, E19, E20, E23, E24, E25, E26, E28, E30, E31, E32, E33) have partial segments of their sequence reported as subsets of other epitopes, whereas E9, E13, E21 are exact match to previously reported sequences. For these epitopes, a comparison showing the overlap between the predicted epitopes in this study and previously known epitopes documented in IEDB is given in Table S5. In addition to the epitopes in the IEDB, we compared our consensus predicted epitopes with the previously reported predictions^62–67^ in Table S6. This comparison shows a varying degree of overlap in the predicted sequences. The novelty results confirm that thirty epitopes have not been previously tested experimentally as LASV epitopes, suggesting that their therapeutic potentials in designing vaccines against LASV can be further explored.

## Conclusion

LASV hemorrhagic fever is endemic in West Africa, and no approved effective therapeutics are currently available. Therefore, there is an urgent need for the discovery and development of potential antiviral therapeutics. The LASV GP spike has emerged as a promising selective target for the development of novel vaccines as it plays an essential role in the virus-host interaction. Several in-silico studies^62–67^ were performed to predict LASV GP epitopes with the use of a single prediction tool for each type of epitope. We have identified new T and B-cell epitopes using a variety of computational approaches, including twelve epitope prediction methods, protein-peptide docking, and MD simulations. The MHC I and II T-cell epitopes were separately predicted with the LASV GP sequence using well-known prediction methods. The predicted MHC I T-cell epitopes then were prioritized based on the consensus score, binding affinity, and antigenicity, while MHC II T and B-cell epitopes were prioritized based on the consensus score. Novelty analysis of the consensus-selected 33 epitopes showed that thirty of these predicted epitopes have either no overlap or only a partial overlap to previously reported sequences. Within this list of new epitopes, six sequences have no overlap with any known experimentally tested epitopes in the IEDB. In addition, docking and MD simulations were performed to further validate the MHC I T-cell epitopes. The simulation results show that the allele-MHC-I epitopes are stable, with favorable hydrogen-bond and interaction energy. Of these, Epitope E3 (^233^FSRPSPIGY^241^) segment was found to be especially stable. This study demonstrates that the adopted consensus epitope prediction strategy is valuable for in-silico investigations of known epitopes and the identification of new epitopes. Experimental validation of these epitopes may lead to the design and development of effective LASV vaccines.

## Supplementary information

Supplementary Information.

## References

[1] Radoshitzky SR (2019). ICTV Virus Taxonomy Profile: Arenaviridae. J Gen Virol.

[2] Frame JD, Baldwin JM, Gocke DJ, Troup JM (1970). Lassa fever, a new virus disease of man from West Africa. I. Clinical description and pathological findings. Am J Trop Med Hyg.

[3] Gibb R, Moses LM, Redding DW, Jones KE (2017). Understanding the cryptic nature of Lassa fever in West Africa. Pathog Glob Health.

[4] Hastie KM (2017). Structural basis for antibody-mediated neutralization of Lassa virus. Science.

[5] Buckley SM, Casals J (1970). Lassa fever, a new virus disease of man from West Africa. 3. Isolation and characterization of the virus. Am J Trop Med Hyg.

[6] McCormick JB (1987). A case-control study of the clinical diagnosis and course of Lassa fever. J Infect Dis.

[7] Buchmeier MJ, Oldstone MB (1979). Protein structure of lymphocytic choriomeningitis virus: evidence for a cell-associated precursor of the virion glycopeptides. Virology.

[8] Sogoba N, Feldmann H, Safronetz D (2012). Lassa fever in West Africa: evidence for an expanded region of endemicity. Zoonoses Public Health.

[9] Wolff, S. *et al*. Genome Sequence of Lassa Virus Isolated from the First Domestically Acquired Case in Germany. *Genome Announc* **4**, 10.1128/genomeA.00938-16 (2016). 10.1128/genomeA.00938-16 PMC5034122 27660771

[10] Jahrling PB, Peters CJ (1984). Passive antibody therapy of Lassa fever in cynomolgus monkeys: importance of neutralizing antibody and Lassa virus strain. Infect Immun.

[11] Li S (2016). Acidic pH-Induced Conformations and LAMP1 Binding of the Lassa Virus Glycoprotein Spike. PLoS Pathog.

[12] Bederka LH, Bonhomme CJ, Ling EL, Buchmeier MJ (2014). Arenavirus stable signal peptide is the keystone subunit for glycoprotein complex organization. MBio.

[13] Klaus JP (2013). The intracellular cargo receptor ERGIC-53 is required for the production of infectious arenavirus, coronavirus, and filovirus particles. Cell Host Microbe.

[14] Robinson JE (2016). Most neutralizing human monoclonal antibodies target novel epitopes requiring both Lassa virus glycoprotein subunits. Nat Commun.

[15] Nielsen, M., Lundegaard, C. & Lund, O. J. B. b. Prediction of MHC class II binding affinity using SMM-align, a novel stabilization matrix alignment method. **8**, 238 (2007). 10.1186/1471-2105-8-238 PMC1939856 17608956

[16] Yang X, Yu X (2009). An introduction to epitope prediction methods and software. Rev Med Virol.

[17] Zhang L (2018). Multi-epitope vaccines: a promising strategy against tumors and viral infections. Cell Mol Immunol.

[18] Shey RA (2019). In-silico design of a multi-epitope vaccine candidate against onchocerciasis and related filarial diseases. Sci Rep.

[19] Pickett BE (2012). Virus pathogen database and analysis resource (ViPR): a comprehensive bioinformatics database and analysis resource for the coronavirus research community. Viruses.

[20] Sievers F (2011). Fast, scalable generation of high-quality protein multiple sequence alignments using Clustal. Omega..

[21] Berman HM (2000). The Protein Data Bank. Nucleic Acids Res.

[22] Jo S, Kim T, Iyer VG, Im W (2008). CHARMM-GUI: a web-based graphical user interface for CHARMM. J Comput Chem.

[23] Brooks BR (2009). CHARMM: the biomolecular simulation program. J Comput Chem.

[24] Lee J (2016). CHARMM-GUI Input Generator for NAMD, GROMACS, AMBER, OpenMM, and CHARMM/OpenMM Simulations Using the CHARMM36 Additive Force Field. Journal of Chemical Theory and Computation.

[25] Jespersen MC, Peters B, Nielsen M, Marcatili PJNAR (2017). BepiPred-2.0: improving sequence-based B-cell epitope prediction using conformational epitopes..

[26] EL-Manzalawy, Y. & Dobbs, D. & Honavar, V. Predicting linear B-cell epitopes using string kernels. *J Mol Recognit.* **21**, 243–255 (2008). 10.1002/jmr.893 PMC2683948 18496882

[27] Saha, S. & Raghava, G. In *International Conference on Artificial Immune Systems*. 197–204 (Springer).

[28] Ponomarenko, J. *et al*. ElliPro: a new structure-based tool for the prediction of antibody epitopes. **9**, 514 (2008). 10.1186/1471-2105-9-514 PMC2607291 19055730

[29] Rubinstein, N. D., Mayrose, I., Martz, E. & Pupko, T. J. B. b. Epitopia: a web-server for predicting B-cell epitopes. **10**, 287 (2009). 10.1186/1471-2105-10-287 PMC2751785 19751513

[30] Kringelum, J. V., Lundegaard, C., Lund, O. & Nielsen, M. J. P. C. B. Reliable B cell epitope predictions: impacts of method development and improved benchmarking. **8**, e1002829 (2012). 10.1371/journal.pcbi.1002829 PMC3531324 23300419

[31] Singh H, Raghava GP (2003). ProPred1: prediction of promiscuous MHC Class-I binding sites. Bioinformatics.

[32] Bhasin M, Raghava GJV (2004). Prediction of CTL epitopes using QM. SVM and ANN techniques..

[33] Larsen MV (2007). Large-scale validation of methods for cytotoxic T-lymphocyte epitope prediction. BMC Bioinformatics.

[34] Vita R (2015). The immune epitope database (IEDB) 3.0. Nucleic Acids Res.

[35] Singh H, Raghava GP (2001). ProPred: prediction of HLA-DR binding sites. Bioinformatics.

[36] Jensen KK (2018). Improved methods for predicting peptide binding affinity to MHC class II molecules. Immunology.

[37] Dimitrov, I., Garnev, P., Flower, D. R. & Doytchinova, I. J. B. EpiTOP—a proteochemometric tool for MHC class II binding prediction. **26**, 2066–2068 (2010). 10.1093/bioinformatics/btq324 20576624

[38] Doytchinova IA, Flower DR (2007). VaxiJen: a server for prediction of protective antigens, tumour antigens and subunit vaccines. BMC Bioinformatics.

[39] Liu, J., Chen, K. Y. & Ren, E. C. J. E. J. O. I. Structural insights into the binding of hepatitis B virus core peptide to HLA-A2 alleles: Towards designing better vaccines. **41**, 2097–2106 (2011). 10.1002/eji.201041370 21538979

[40] Hourigan CS (2006). The structure of the human allo-ligand HLA-B*3501 in complex with a cytochrome p450 peptide: steric hindrance influences TCR allo-recognition. Eur J Immunol.

[41] Waterhouse A (2018). SWISS-MODEL: homology modelling of protein structures and complexes. Nucleic Acids Res.

[42] Bienert, S. *et al*. The SWISS-MODEL Repository—new features and functionality. **45**, D313–D319 (2016). 10.1093/nar/gkw1132 PMC5210589 27899672

[43] Guex N, Peitsch MC, Schwede T (2009). Automated comparative protein structure modeling with SWISS-MODEL and Swiss-PdbViewer: a historical perspective. Electrophoresis.

[44] Morris, G. M. *et al*. AutoDock4 and AutoDockTools4: Automated docking with selective receptor flexibility. **30**, 2785–2791 (2009). 10.1002/jcc.21256 PMC2760638 19399780

[45] Trott O, Olson AJ (2010). AutoDock Vina: improving the speed and accuracy of docking with a new scoring function, efficient optimization, and multithreading. J Comput Chem.

[46] Huang J, MacKerell AD (2013). CHARMM36 all-atom additive protein force field: validation based on comparison to NMR data. J Comput Chem.

[47] Phillips JC (2005). Scalable molecular dynamics with NAMD. J Comput Chem.

[48] Essmann, U. *et al*. A smooth particle mesh Ewald method. **103**, 8577–8593 (1995).

[49] Ryckaert, J.-P., Ciccotti, G. & Berendsen, H. J. J. J. O. C. P. Numerical integration of the Cartesian equations of motion of a system with constraints: molecular dynamics of n-alkanes. **23**, 327–341 (1977).

[50] Brooks, M. M., Hallstrom, A. & Peckova, M. J. S. I. M. A simulation study used to design the sequential monitoring plan for a clinical trial. **14**, 2227–2237 (1995). 10.1002/sim.4780142006 8552899

[51] Humphrey W, Dalke A, Schulten K (1996). VMD: visual molecular dynamics. J Mol Graph.

[52] Borley, D. W. *et al*. Evaluation and use of in-silico structure-based epitope prediction with footand-mouth disease virus. **8**, e61122 (2013). 10.1371/journal.pone.0061122 PMC3646828 23667434

[53] Freire MC (2017). Mapping Putative B-Cell Zika Virus NS1 Epitopes Provides Molecular Basis for Anti-NS1 Antibody Discrimination between Zika and Dengue. Viruses..

[54] Schumacher TN (1991). Peptide selection by MHC class I molecules. Nature.

[55] Nielsen M, Lund O, Buus S, Lundegaard C (2010). MHC class II epitope predictive algorithms. Immunology.

[56] Sanchez-Trincado JL, Gomez-Perosanz M, Reche PA (2017). Fundamentals and Methods for Tand B-Cell Epitope Prediction. J Immunol Res.

[57] Ahmad B, Ashfaq UA, Rahman MU, Masoud MS, Yousaf MZ (2019). Conserved B and T cell epitopes prediction of ebola virus glycoprotein for vaccine development: An immunoinformatics approach. Microb Pathog.

[58] Benkert P, Biasini M, Schwede T (2011). Toward the estimation of the absolute quality of individual protein structure models. Bioinformatics.

[59] Ramachandran GN, Ramakrishnan C, Sasisekharan V (1963). Stereochemistry of polypeptide chain configurations. J Mol Biol.

[60] Colovos C, Yeates TO (1993). Verification of protein structures: patterns of nonbonded atomic interactions. Protein Sci.

[61] Antunes DA (2010). Structural allele-specific patterns adopted by epitopes in the MHC-I cleft and reconstruction of MHC: peptide complexes to cross-reactivity assessment. PLoS One.

[62] Verma SK, Yadav S, Kumar A (2015). In silico prediction of B- and T- cell epitope on Lassa virus proteins for peptide based subunit vaccine design. Adv Biomed Res.

[63] Faisal AM, Imtiaz SH, Zerin T, Rahman T, Shekhar HU (2017). Computer aided epitope design as a peptide vaccine component against Lassa virus. Bioinformation.

[64] Wauquier N (2019). HLA-C-restricted viral epitopes are associated with an escape mechanism from KIR2DL2(+) NK cells in Lassa virus infection. EBioMedicine.

[65] Hossain MU (2018). Design of peptide-based epitope vaccine and further binding site scrutiny led to groundswell in drug discovery against Lassa virus. 3 Biotech.

[66] Botten J (2006). Identification of protective Lassa virus epitopes that are restricted by HLA-A2. J Virol.

[67] Boesen A, Sundar K, Coico R (2005). Lassa fever virus peptides predicted by computational analysis induce epitope-specific cytotoxic-T-lymphocyte responses in HLA-A2.1 transgenic mice. Clin Diagn Lab Immunol.

