# Supplementary material for: In-silico identification of the vaccine candidate epitopes against the Lassa virus hemorrhagic fever
Source: Sci Rep. 2020 May 6;10:7667. doi: 10.1038/s41598-020-63640-1 (PMC7203123; doi:10.1038/s41598-020-63640-1)
Supplement: Supplementary file 1 — Supplementary Information. [file 41598_2020_63640_MOESM1_ESM.docx]

**Supplementary Information**

***In-silico* identification of the vaccine candidate epitopes against the Lassa virus hemorrhagic fever**

Prabin Baral^a#^, Elumalai Pavadai^a,#,§^, Bernard S. Gerstman^a,b^ and Prem P. Chapagain^a,b^*

aDepartment of Physics, Florida International University, Miami, Florida 33199, USA

bBiomolecular Science Institute, Florida International University, Miami, Florida 33199, USA

^#^ Equal contribution

^§^Current address: Department of Physiology and Biophysics, Boston University School of Medicine, Boston MA 02118, USA

* Corresponding Author, Email: chapagap@fiu.edu

**Table S1**. MHC class I epitopes prediction

| Propred-1 | CTLPred | NetCTL 1.2 |
| --- | --- | --- |
| IEEVMNIVLI | TFFQEVPHV | TTSLYKGVY |
| VMNIVLIALSVLAVLKGL | EVPHVIEEV | CTKNNSHHY |
| VMNIVLIAL | VPHVIEEVM | ITEMLQKEY |
| NIVLIALSV | VIEEVMNIV | VLAVLKGLY |
| IALSVLAVL | LIALSVLAV | LSIPNFNQY |
| SVLAVLKGL | AVLKGLYNF | NTSIINHKF |
| FATCGLVGLVTFL | FATCGLVGL | MTSYQYLII |
| FATCGLVGL | VYELQTLEL | LSQRTRDIY |
| GLVGLVTFL | KNNSHHYIM | NWDCIMTSY |
| NETGLELTL | IINHKFCNL | FSRPSPIGY |
| IINHKFCNL | ALMSIISTF | IMCIPYCNY |
| LSDAHKKNL | MSIISTFHL | LRDIMCIPY |
| SRPSPIGYL | QYNLSHSYA | QADNMITEM |
| RRGTFTWTL | GVLQTFMRM | GRSCTTSLY |
| LIEAELKCF | MAWGGSYIA | MRMAWGGSY |
|  | AWGGSYIAL | YCNYSKYWY |
|  | FSRPSPIGY | NMETLNMTM |
|  | SQRTRDIYI | VQYNLSHSY |
|  | RTRDIYISR |  |
|  | RRGTFTWTL |  |
|  | RWMLIEAEL |  |
|  | ELKCFGNTA |  |
|  | MLRLFDFNK |  |
|  | FNKQAIQRL |  |
|  | NALINDQLI |  |
|  | IPYCNYSKY |  |

**Table S2.** MHC class II epitopes prediction (The core epitopes predicted by NetMHCII2.3 are highlighted in bold).

| ProPred | NetMHCII2.3 | EpiTOP3.0 |
| --- | --- | --- |
| MGQIVTFFQ | IALSV**LAVLKGLYN**F | MGQIVTFFQ |
| FFQEVPHVI | ALSV**LAVLKGLYN**FA | FQEVPHVIE |
| VMNIVLIALSVL | LSV**LAVLKGLYN**FAT | IEEVMNIVL |
| MNIVLIALSVLAV | SV**LAVLKGLYN**FATC | VLIALSVLA |
| IVLIALSVL | V**LAVLKGLYN**FATCG | LIALSVLAV |
| LKGLYNFATCG | **LAVLKGLYN**FATCGL | LSVLAVLKG |
| LVGLVTFLLLCGRSC | DCIMTS**YQYLIIQNT** | LAVLKGLYN |
| VYELQTLEL | CIMTS**YQYLIIQNT**T | LKGLYNFAT |
| LNMTMPLSC | IMTS**YQYLIIQNT**TW | FATCGLVGL |
| LELTLTNTSII | TS**YQYLIIQNT**TWED | LCGRSCTTS |
| INHKFCNLS | RPSPIG**YLGLLSQR**T | LYKGVYELQ |
| MSIISTFHLSI | PSPIG**YLGLLSQRT**R | YKGVYELQT |
| MSIISTFHL | SPIG**YLGLLSQRT**RD | VYELQTLEL |
| FNQYEAMSC | PIG**YLGLLSQRT**RDI | YELQTLELN |
| VQYNLSHSY | IG**YLGLLSQRT**RDIY | LQTLELNME |
| ISVQYNLSH | G**YLGLLSQRT**RDIYI | LELNMETLN |
| YNLSHSYAG | MSIIST**FHLSIPNFN** | LNMETLNMT |
| YAGDAANHC | SIIST**FHLSIPNFN**Q | LNMTMPLSC |
| MRMAWGGSY | IIST**FHLSIPNFN**QY | IMVGNETGL |
| LQTFMRMAW | IST**FHLSIPNFN**QYE | MVGNETGLE |
| MTSYQYLII | ST**FHLSIPNFN**QYEA | VGNETGLEL |
| YQYLIIQNT | T**FHLSIPNFN**QYEAM | LELTLTNTS |
| LIIQNTTWE | GVLQTF**MRMAWGGSY** | LTNTSIINH |
| IGYLGLLSQ | VLQTF**MRMAWGGSY**I | IINHKFCNL |
| LLSQRTRDI | LQTF**MRMAWGGSY**IA | INHKFCNLS |
| IYISRRRRG | QTF**MRMAWGGSY**IAL | LSDAHKKNL |
| WMLIEAELK | TF**MRMAWGGSY**IALD | LYDHALMSI |
| LKCFGNTAV | F**MRMAWGGSY**IALDS | LMSIISTFH |
| LRLFDFNKQ | LSVLAV**LKGLYNFAT** | MSIISTFHL |
| IQRLKAPAQ | SVLAV**LKGLYNFAT**C | IISTFHLSI |
| IQLINKAVN | VLAV**LKGLYNFAT**CG | FHLSIPNFN |
| LINDQLIMK | LAV**LKGLYNFAT**CGL | FNQYEAMSC |
| LIMKNHLRDIM | AV**LKGLYNFAT**CGLV | MSCDFNGGK |
| LRDIMCIPY | V**LKGLYNFAT**CGLVG | FNGGKISVQ |
| YCNYSKYWY | VGLVT**FLLLCGRSC**T | ISVQYNLSH |
| WYLNHTTTGR | GLVT**FLLLCGRSC**TT | LSHSYAGDA |
| LVSNGSYLN | LVT**FLLLCGRSC**TTS | VLQTFMRMA |
|  | VT**FLLLCGRSC**TTSL | FMRMAWGGS |
|  | NNSHH**YIMVGNETG**L | MRMAWGGSY |
|  | NSHH**YIMVGNETG**LE | WGGSYIALD |
|  | SHH**YIMVGNETG**LEL | LDSGCGNWD |
|  | HH**YIMVGNETG**LELT | WDCIMTSYQ |
|  | HKKNL**YDHALMSII**S | LIIQNTTWE |
|  | KKNL**YDHALMSII**ST | WEDHCQFSR |
|  | KNL**YDHALMSII**STF | FSRPSPIGY |
|  | NL**YDHALMSII**STFH | IGYLGLLSQ |
|  | L**YDHALMSII**STFHL | LLSQRTRDI |
|  | GVLQT**FMRMAWGGS**Y | LSQRTRDIY |
|  | VLQT**FMRMAWGGS**YI | YISRRRRGT |
|  | LQT**FMRMAWGGS**YIA | ISRRRRGTF |
|  | QT**FMRMAWGGS**YIAL | WMLIEAELK |
|  | T**FMRMAWGGS**YIALD | MLIEAELKC |
|  | AQT**SIQLINKAV**NAL | LIEAELKCF |
|  | QT**SIQLINKAV**NALI | IEAELKCFG |
|  | T**SIQLINKAV**NALIN | FGNTAVAKC |
|  | QTSIQL**INKAVNALI** | FCDMLRLFD |
|  | TSIQL**INKAVNALI**N | FDFNKQAIQ |
|  | SIQL**INKAVNALI**ND | FNKQAIQRL |
|  | IQL**INKAVNALI**NDQ | LKAPAQTSI |
|  | QL**INKAVNALI**NDQL | IQLINKAVN |
|  | SQRTRD**IYISRRRRG** | LINKAVNAL |
|  | QRTRD**IYISRRRRG**T | LINDQLIMK |
|  | RTRD**IYISRRRRG**TF | LIMKNHLRD |
|  | TRD**IYISRRRRG**TFT | IMKNHLRDI |
|  | RD**IYISRRRRG**TFTW | LRDIMCIPY |
|  | D**IYISRRRRG**TFTWT | WYLNHTTTG |
|  | **IYISRRRRG**TFTWTL | LNHTTTGRT |
|  | NLYDHA**LMSIISTFH** | WLVSNGSYL |
|  | LYDHA**LMSIISTFH**L | LVSNGSYLN |
|  | YDHA**LMSIISTFH**LS | FSDDIEQQA |
|  | DHA**LMSIISTFH**LSI | ITEMLQKEY |
|  | HA**LMSIISTFH**LSIP | MLQKEYMER |
|  | MTSYQ**YLIIQNTTW**E | |
|  | TSYQ**YLIIQNTTW**ED | |
|  | SYQ**YLIIQNTTW**EDH | |
|  | YQ**YLIIQNTTW**EDHC | |
|  | Q**YLIIQNTTW**EDHCQ | |
|  | LIALS**VLAVLKGLY**N | |
|  | IALS**VLAVLKGLY**NF | |
|  | ALS**VLAVLKGLY**NFA | |
|  | LS**VLAVLKGLY**NFAT | |
|  | VANGV**LQTFMRMAW**G | |
|  | ANGV**LQTFMRMAW**GG | |
|  | NGV**LQTFMRMAW**GGS | |
|  | GV**LQTFMRMAW**GGSY | |
|  | V**LQTFMRMAW**GGSYI | |
|  | DFNGG**KISVQYNLS**H | |
|  | FNGG**KISVQYNLS**HS | |
|  | NGG**KISVQYNLS**HSY | |
|  | GG**KISVQYNLS**HSYA | |
|  | G**KISVQYNLS**HSYAG | |
|  | NETGLE**LTLTNTSII** | |
|  | ETGLE**LTLTNTSII**N | |
|  | TGLE**LTLTNTSII**NH | |
|  | GLE**LTLTNTSII**NHK | |
|  | LE**LTLTNTSII**NHKF | |
|  | LYDHAL**MSIISTFHL** | |
|  | YDHAL**MSIISTFHL**S | |
|  | DHAL**MSIISTFHL**SI | |
|  | HAL**MSIISTFHL**SIP |  |
|  | AL**MSIISTFHL**SIPN |  |
|  | L**MSIISTFHL**SIPNF |  |

**Table S3.** Comparison of B-cell epitopes (this work) with the Robinson et al.’s^1^ B-cell epitopes. The common sequence between these two epitopes is highlighted in red color. Here, LASV-I, LASV-II and LASV-III represent different lineages from Nigeria while LASV-IV represents those from Sierra Leone^1^. The blue colors represent the amino acids differing in lineages.

| Epitope | Sequence | | Interval  This-work (Robinson) | | Sequence (ref. Robinson) | |
| --- | --- | --- | --- | --- | --- | --- |
| E24 | LSDAHKKNLYD | | 120-130 (119-134) | | NLSDAHKKNLYDHALM (LASV-I, II, IV) | |
|  |  | |  | | NLSDAHKKNLYDH**T**LM (LASV-III) | |
| E30 | SDSEGKDTPG | | 267-276 (270-278) | | EGKDTPGGY (LASV IV) | |
|  |  | |  | | EG**NE**TPGGY (LASV I-III) | |
| E31 | NHTTTGRT | | 373-380 (369-373) | | YWYLN (LASV I-IV) | |
| E32 | ETHFSDDIE | | 396-404 (401-415) | | DDIEQQADNMITEML (LASV I, III, IV) | |
|  |  | |  | | DDIEQQADNMITE**L**L (LASV II) | |
| E33 | MLQKEYMERQ | | 414-423 (401-415) | | DDIEQQADNMITEML (LASV I, III, IV) | |
|  | |  | |  | | DDIEQQADNMITE**L**L (LASV II) |

**Table S4.** Intermolecular hydrogen bond pairs between allele and epitope.

| **A1::E1** | | |
| --- | --- | --- |
| donor | acceptor | occupancy |
| THR40-Side | THR97-Side | 7.10% |
| CYS41-Side | HSD138-Side | 1.70% |
| LEU46-Main | ALA174-Main | 1.10% |
| LEU46-Main | LYS170-Main | 2.20% |
| GLN179-Side | THR40-Side | 5.35% |
| GLN179-Side | THR40-Main | 8.15% |
| THR40-Side | GLN179-Side | 2.15% |
| GLY42-Main | GLN179-Side | 1.95% |
| THR97-Side | LEU43-Main | 9.00% |
| TYR183-Side | ALA39-Main | 2.00% |
|  |  |  |
|  |  |  |
| **A2::E2** |  |  |
| donor | acceptor | occupancy |
| ASN114-Side | GLU152-Side | 1.25% |
| ASN119-Side | GLU63-Side | 65.00% |
| ASN119-Side | TYR59-Main | 29.70% |
| THR73-Side | ILE113-Main | 2.30% |
| HSD70-Side | HSD115-Side | 16.25% |
| THR73-Side | HSD115-Side | 76.55% |
| HSD115-Side | GLU152-Side | 10.85% |
|  |  |  |
|  |  |  |
| **A3::E3** |  |  |
| donor | acceptor | occupancy |
| TYR241-Side | ASN63-Side | 21.05% |
| ARG62-Side | PRO238-Main | 3.65% |
| PHE233-Main | SER77-Side | 76.65% |
| ARG235-Side | ALA150-Main | 24.15% |
| ARG97-Side | SER234-Side | 18.35% |
| ARG62-Side | SER237-Side | 20.50% |
| TYR241-Main | TYR159-Side | 1.35% |
| ARG62-Side | GLY240-Main | 10.45% |
| TYR241-Side | TYR171-Side | 2.90% |
| ASN63-Side | TYR241-Side | 1.60% |
| SER234-Side | TRP147-Side | 8.00% |
| TYR241-Side | TYR59-Side | 1.25% |
| TYR7-Side | TYR241-Side | 2.90% |
| ASN70-Side | TYR241-Main | 6.70% |
| TYR241-Side | TYR7-Side | 3.15% |
| TYR59-Side | TYR241-Side | 4.35% |
| TRP167-Side | ILE239-Main | 15.85% |
| TYR159-Side | ILE239-Main | 16.45% |
| TYR171-Side | TYR241-Side | 1.60% |
|  |  |  |
|  |  |  |
|  |  |  |
| **A4 ::E4** |  |  |
| donor | acceptor | occupancy |
| THR261-Main | HSD94-Side | 6.25% |
| ARG259-Side | THR97-Side | 1.98% |
| TRP264-Side | ALA174-Main | 66.14% |
| TYR183-Side | THR261-Side | 5.69% |
| PHE262-Main | GLN138-Side | 3.25% |
| THR261-Side | TYR183-Side | 3.41% |
| TRP171-Side | LEU266-Main | 13.22% |
| ARG258-Main | THR97-Side | 8.64% |
| ARG258-Side | GLU100-Side | 18.05% |
| HSD94-Side | ARG258-Main | 1.32% |
| ARG259-Side | ASN90-Side | 2.14% |
| ARG259-Side | GLN86-Side | 2.34% |
| ARG259-Side | HSD94-Side | 3.30% |
| ARG259-Side | GLU87-Side | 5.44% |
| ARG258-Main | HSD94-Side | 9.20% |
|  |  |  |

| **Table S5:** New Epitopes and the peptides in which they were reported in IEDB. The common sequences are highlighted in red. | | | | |  | |
| --- | --- | --- | --- | --- | --- | --- |
| Epitope | Sequence (this work) | | IEDB Reported peptides | | References | |
| E2 | IINHKFCNL | | SIINHKFCNL | | 2, 3 | |
|  |  | | SIINHKFCNLSDAHK | | 3 | |
|  |  | | TLTNTSIINHKFCNL | | 3 | |
| E3 | FSRPSPIGY | | CQFSRPSPIGYLGLL | | 3 | |
| E5 | MGQIVTFFQ | | MGQIVTFFQEVPHVI | | 3 | |
| E6 | VYELQTLEL | | LYKGVYELQTLELNM | | 3 | |
| E7 | LNMTMPLSC | | ETLNMTMPLSCTKNN | | 3 | |
| E8 | INHKFCNLS | | SIINHKFCNLSDAHK | | 3 | |
| E9 | | | MSIISTFHL | | LMSIISTFHL 3, 4 | |
|  |  | | MSIISTFHL | | 3 | |
|  |  | | HALMSIISTFHLSIP | | 3 | |
| E10 | FNQYEAMSC | | HLSIPNFNQYEAMSC | | 3 | |
|  |  | | NFNQYEAMSCDFNGG | |  | |
| E11 | ISVQYNLSH | | DFNGGKISVQYNLSH | | 3 | |
|  |  | | KISVQYNLSHSYAVD | |  | |
|  |  | | KISVQYNLSH | |  | |
| E12 | LQTFMRMAW | | GVLQTFMRMAWGGSY | | 3 | |
| E13 | | | MRMAWGGSY | | MRMAWGGSY 3, 5 | |
|  | | |  | | FMRMAWGGSY 3, 5 | |
|  | | |  | | FMRMAWGGSYIALDS 4 | |
|  | | |  | | GVLQTFMRMAWGGSY 4 | |
| E14 | | | YQYLIIQNT | | MRMAWGGSYI 3, 5  DCIMTSYQYLIIQNT 3 | |
|  |  | | SYQYLIIQNTTWEDH | |  | |
| E15 | LIIQNTTWE | | SYQYLIIQNTTWEDH | | 3 | |
| E16 | IGYLGLLSQ | | PSPIGYLGLLSQRTR | | 3, 6 | |
| E17 | LLSQRTRDI | | YLGLLSQRTRDIYIS | | 3, 6 | |
| E19 | WMLIEAELK | | RWMLIEAELKCFG | | 3, 7 | |
|  |  | | TRWMLIEAELKCFGN | | 3 | |
|  |  | | PGGYCLTRWMLIEAELKCFG | | 3 | |
|  |  | | RWMLIEAELKCFGNTAVAKC | | 3, 7 | |
| E20 | IQLINKAVN | | QMSIQLINKAVNALI | | 3 | |
| E21 | LINDQLIMK | | LINDQLIMK | | 3 | |
|  |  | | VNALINDQLIMKNHL | |  | |
| E23 | LVSNGSYLN | | SLPKCWLVSNGSYLN | | 3 | |
|  |  | | WLVSNGSYLNETHFS | |  | |
| E24 | LSDAHKKNLYD | | KFCNLSDAHKKNLYD | | 3 | |
|  |  | | SDAHKKNLY | |  | |
| E25 | PNFNQYEA | | HLSIPNFNQYEAMSC | | 3 | |
| E26 | DFNGGKI | | DFNGGKISVQYNLSH | | 3 | |
|  |  | | EAMSCDFNGGKISVQ | |  | |
| E28 | LDSGCGNWDCIMTSYQY | | DCIMTSYQY | | 3 | |
| E32 | ETHFSDDIE | | LNETHFSDDIEQQ | | 3, 7 | |
|  |  | | ETHFSDDIEQQADNM | | 3 | |
|  |  | | GSYLNETHFSDDIEQ  LVSNGSYLNETHFSDDIEQQ  LNETHFSDDIEQQADNMITE | | 3  3, 7  3, 7 | |
| E33 | MLQKEYMERQ | | MLQKEYMER | | 3 | |
|  |  | | ITEMLQKEYMERQGK | |  | |
|  |  | |  | |  | |

**Table S6: Comparison of epitopes with already reported epitopes in** literature. The common sequences are highlighted in red.

| Epitope | Sequence (this work) | Previously reported | References |
| --- | --- | --- | --- |
| E1 | FATCGLVGL | GLYNFATCGL | 4 |
|  |  | GLVGLVTFL | 4, 8 |
| E2 | IINHKFCNL | IMVGNETGLELTLTNTSIIN | 9 |
|  |  | MVGNETGLELTLTNTSIINH | 9 |
|  |  | NLSDAHKKNLYDHAL | 10 |
| E3 | FSRPSPIGY | PSPIGYLGLLSQRTR | 6 |
|  |  | YLGLLSQRTRDIYIS |  |
| E4 | RRGTFTWTL | GTFTWTLSDSEGKDTPGGYC | 9,11 |
|  |  | LLGTFTWTLSDSEGKDTPGG |  |
|  |  | LLGTFTWTL | 11 |
| E6 | VYELQTLEL | SLYKGVYEL | 4, 8 |
| E7 | LNMTMPLSC | NMETLNMTMPLSCTKNNSHH | 9 |
|  |  | MTMPLSCTK | 12 |
| E8 | INHKFCNLS | IMVGNETGLELTLTNTSIIN | 9 |
|  |  | NLSDAHKKNLYDHAL | 10 |
| E9 | MSIISTFHL | NLYDHALMSI | 4 |
|  |  | LMSIISTFHL |  |
|  |  | IISTFHLSI |  |
| E10 | FNQYEAMSC | NQYEAMSCDFNGGKISVQYN | 9 |
| E11 | ISVQYNLSH | NQYEAMSCDFNGGKISVQYN | 9 |
|  |  | VQYNLSHSY | 10, 12 |
| E12 | LQTFMRMAW | RMAWGGSYI | 4 |
| E13 | MRMAWGGSY | MAWGGSYIA | 4 |
|  |  | RMAWGGSYI |  |
| E14 | YQYLIIQNT | LDSGRGNWDCIMTSYQYLII | 9 |
|  |  | GNWDCIMTSYQYLIIQNTTW | 9 |
|  |  | IMTSYQYLI | 4 |
|  |  | CIMTSYQYL | 8 |
| E15 | LIIQNTTWE | LDSGRGNWDCIMTSYQYLII | 9 |
|  |  | GNWDCIMTSYQYLIIQNTTW | 9 |
|  |  | IMTSYQYLI | 4 |
|  |  | CIMTSYQYL | 8 |
| E16 | IGYLGLLSQ | PSPIGYLGLLSQRTR | 6 |
|  |  | YLGLLSQRTRDIYIS |  |
| E17 | LLSQRTRDI | PSPIGYLGLLSQRTR | 6 |
|  |  | YLGLLSQRTRDIYIS |  |
| E18 | IYISRRRRG | GTFTWTLSDSEGKDTPGGYC | 9 |
|  |  | YLGLLSQRTRDIYIS | 6 |
| E19 | WMLIEAELK | RWMLIEAEL | 11 |
|  |  | RWMLIEAELKCFGNTAVAKC | 7 |
|  |  | ELKCFGNTAVAKCNEKHDEE | 7 |
| E20 | IQLINKAVN | AEAQMSIQLINKAVNA | 11, 8 |
|  |  | LINKAVNAL | 8 |
| E21 | LINDQLIMK | ALINDQLIM | 4 |
|  |  | LINKAVNAL | 8 |
| E23 | LVSNGSYLN | WLVSNGSYL | 8 |
|  |  | LVSNGSYLNETHFSDDIEQQ | 7 |
|  |  | LNETHFSDDIEQQADNMITE | 7 |
| E24 | LSDAHKKNLYD | NLYDHALMSI | 4 |
|  |  | NLSDAHKKNLYDHAL | 4, 10 |
| E25 | PNFNQYEA | NQYEAMSCDFNGGKISVQYN | 9 |
| E26 | DFNGGKI | NQYEAMSCDFNGGKISVQYN | 9 |
| E27 | LSHSYAGDAANHCGT | VQYNLSHSY | 12 |
|  |  | VEYNLSHSYAGDA | 10 |
| E28 | LDSGCGNWDCIMTSYQY | IMTSYQYLI | 4 |
|  |  | CIMTSYQYL | 8 |
| E29 | ISRRRRGT | GTFTWTLSDSEGKDTPGGYC | 9 |
|  |  | YLGLLSQRTRDIYIS | 6 |
| E30 | SDSEGKDTPG | GTFTWTLSDSEGKDTPGGYC | 9 |
|  |  | PGGYCLTRWMLIEAELKCFG | 7 |
| E32 | ETHFSDDIE | LVSNGSYLNETHFSDDIEQQ | 7 |
|  |  | LNETHFSDDIEQQADNMITE |  |
| E33 | MLQKEYMERQ | YMERQGKTPL | 5 |
|  |  |  |  |


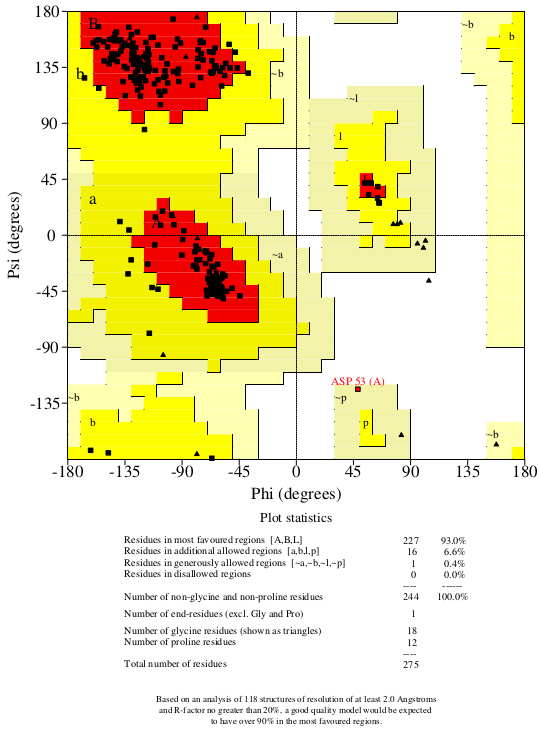


**Figure S1:** Ramachandran plot of the A4 model (generated by using PROCHECK^13,14^).

**References**

1. Robinson, J. E.; Hastie, K. M.; Cross, R. W.; Yenni, R. E.; Elliott, D. H.; Rouelle, J. A.; Kannadka, C. B.; Smira, A. A.; Garry, C. E.; Bradley, B. T.; Yu, H.; Shaffer, J. G.; Boisen, M. L.; Hartnett, J. N.; Zandonatti, M. A.; Rowland, M. M.; Heinrich, M. L.; Martinez-Sobrido, L.; Cheng, B.; de la Torre, J. C.; Andersen, K. G.; Goba, A.; Momoh, M.; Fullah, M.; Gbakie, M.; Kanneh, L.; Koroma, V. J.; Fonnie, R.; Jalloh, S. C.; Kargbo, B.; Vandi, M. A.; Gbetuwa, M.; Ikponmwosa, O.; Asogun, D. A.; Okokhere, P. O.; Follarin, O. A.; Schieffelin, J. S.; Pitts, K. R.; Geisbert, J. B.; Kulakoski, P. C.; Wilson, R. B.; Happi, C. T.; Sabeti, P. C.; Gevao, S. M.; Khan, S. H.; Grant, D. S.; Geisbert, T. W.; Saphire, E. O.; Branco, L. M.; Garry, R. F., Most neutralizing human monoclonal antibodies target novel epitopes requiring both Lassa virus glycoprotein subunits. *Nat Commun* **2016,** *7*, 11544.

2. Kotturi, M. F.; Botten, J.; Sidney, J.; Bui, H. H.; Giancola, L.; Maybeno, M.; Babin, J.; Oseroff, C.; Pasquetto, V.; Greenbaum, J. A.; Peters, B.; Ting, J.; Do, D.; Vang, L.; Alexander, J.; Grey, H.; Buchmeier, M. J.; Sette, A., A multivalent and cross-protective vaccine strategy against arenaviruses associated with human disease. *PLoS Pathog* **2009,** *5* (12), e1000695.

3. Vita, R.; Overton, J. A.; Greenbaum, J. A.; Ponomarenko, J.; Clark, J. D.; Cantrell, J. R.; Wheeler, D. K.; Gabbard, J. L.; Hix, D.; Sette, A.; Peters, B., The immune epitope database (IEDB) 3.0. *Nucleic Acids Res* **2015,** *43* (Database issue), D405-12.

4. Botten, J.; Alexander, J.; Pasquetto, V.; Sidney, J.; Barrowman, P.; Ting, J.; Peters, B.; Southwood, S.; Stewart, B.; Rodriguez-Carreno, M. P.; Mothe, B.; Whitton, J. L.; Sette, A.; Buchmeier, M. J., Identification of protective Lassa virus epitopes that are restricted by HLA-A2. *J Virol* **2006,** *80* (17), 8351-61.

5. Wauquier, N.; Petitdemange, C.; Tarantino, N.; Maucourant, C.; Coomber, M.; Lungay, V.; Bangura, J.; Debre, P.; Vieillard, V., HLA-C-restricted viral epitopes are associated with an escape mechanism from KIR2DL2(+) NK cells in Lassa virus infection. *EBioMedicine* **2019,** *40*, 605-613.

6. Kotturi, M. F.; Botten, J.; Maybeno, M.; Sidney, J.; Glenn, J.; Bui, H. H.; Oseroff, C.; Crotty, S.; Peters, B.; Grey, H.; Altmann, D. M.; Buchmeier, M. J.; Sette, A., Polyfunctional CD4+ T cell responses to a set of pathogenic arenaviruses provide broad population coverage. *Immunome Res* **2010,** *6*, 4.

7. Meulen, J.; Badusche, M.; Satoguina, J.; Strecker, T.; Lenz, O.; Loeliger, C.; Sakho, M.; Koulemou, K.; Koivogui, L.; Hoerauf, A., Old and New World arenaviruses share a highly conserved epitope in the fusion domain of the glycoprotein 2, which is recognized by Lassa virus-specific human CD4+ T-cell clones. *Virology* **2004,** *321* (1), 134-43.

8. Boesen, A.; Sundar, K.; Coico, R., Lassa fever virus peptides predicted by computational analysis induce epitope-specific cytotoxic-T-lymphocyte responses in HLA-A2.1 transgenic mice. *Clin Diagn Lab Immunol* **2005,** *12* (10), 1223-30.

9. Verma, S. K.; Yadav, S.; Kumar, A., In silico prediction of B- and T- cell epitope on Lassa virus proteins for peptide based subunit vaccine design. *Adv Biomed Res* **2015,** *4*, 201.

10. Krasko, A. G.; Moshnikova, A. B.; Kozhich, A. T.; Tchikin, L. D.; Ivanov, V. T.; Vladyko, A. S.; Lukashevich, I. S., Lassa virus glycoproteins: antigenic and immunogenic properties of synthetic peptides to GP1. *Arch Virol* **1990,** *115* (1-2), 133-7.

11. Faisal, A. M.; Imtiaz, S. H.; Zerin, T.; Rahman, T.; Shekhar, H. U., Computer aided epitope design as a peptide vaccine component against Lassa virus. *Bioinformation* **2017,** *13* (12), 417-429.

12. Hossain, M. U.; Omar, T. M.; Oany, A. R.; Kibria, K. M. K.; Shibly, A. Z.; Moniruzzaman, M.; Ali, S. R.; Islam, M. M., Design of peptide-based epitope vaccine and further binding site scrutiny led to groundswell in drug discovery against Lassa virus. *3 Biotech* **2018,** *8* (2), 81.

13. Laskowski, R. A.; MacArthur, M. W.; Moss, D. S.; Thornton, J. M., PROCHECK: a program to check the stereochemical quality of protein structures. *Journal of applied crystallography* **1993,** *26* (2), 283-291.

14. Laskowski, R. A.; Rullmannn, J. A.; MacArthur, M. W.; Kaptein, R.; Thornton, J. M., AQUA and PROCHECK-NMR: programs for checking the quality of protein structures solved by NMR. *J Biomol NMR* **1996,** *8* (4), 477-86.
